# A Deficiency of Herp, an Endoplasmic Reticulum Stress Protein, Suppresses Atherosclerosis in ApoE Knockout Mice by Attenuating Inflammatory Responses

**DOI:** 10.1371/journal.pone.0075249

**Published:** 2013-10-28

**Authors:** Shohei Shinozaki, Tsuyoshi Chiba, Koichi Kokame, Toshiyuki Miyata, Eiji Kaneko, Kentaro Shimokado

**Affiliations:** 1 Geriatrics and Vascular Medicine, Tokyo Medical and Dental University Graduate School, Tokyo, Japan; 2 Information Center, National Institute of Health and Nutrition, Tokyo, Japan; 3 Department of Molecular Pathogenesis, National Cerebral and Cardiovascular Center, Osaka, Japan; Osaka University Graduate School of Medicine, Japan

## Abstract

Herp was originally identified as an endoplasmic reticulum (ER) stress protein in vascular endothelial cells. ER stress is induced in atherosclerotic lesions, but it is not known whether Herp plays any role in the development of atherosclerosis. To address this question, we generated Herp- and apolipoprotein E (apoE)-deficient mice (Herp^−/−^; apoE^−/−^ mice) by crossbreeding Herp^−/−^ mice and apoE^−/−^ mice. Herp was expressed in the endothelial cells and medial smooth muscle cells of the aorta, as well as in a subset of macrophages in the atherosclerotic lesions in apoE^−/−^ mice, while there was no expression of Herp in the Herp^−/−^; apoE^−/−^ mice. The doubly deficient mice developed significantly fewer atherosclerotic lesions than the apoE^−/−^ mice at 36 and 72 weeks of age, whereas the plasma levels of cholesterol and triglycerides were not significantly different between the strains. The plasma levels of non-esterified fatty acids were significantly lower in the Herp^−/−^; apoE^−/−^ mice when they were eight and 16 weeks old. The gene expression levels of ER stress response proteins (GRP78 and CHOP) and inflammatory cytokines (IL-1β, IL-6, TNF-α and MCP-1) in the aorta were significantly lower in Herp^−/−^; apoE^−/−^ mice than in apoE^−/−^ mice, suggesting that Herp mediated ER stress-induced inflammation. In fact, peritoneal macrophages isolated from Herp-deficient mice and RAW264.7 macrophages in which Herp was eliminated with a siRNA expressed lower levels of mRNA for inflammatory cytokines when they were treated with tunicamycin. Herp deficiency affected the major mediators of the unfolded protein response, including IRE1 and PERK, but not ATF6. These findings suggest that a deficiency of Herp suppressed the development of atherosclerosis by attenuating the ER stress-induced inflammatory reactions.

## Introduction

There has been an increasing number of reports on endoplasmic reticulum (ER) stress in atherosclerotic lesion. Markers of ER stress and activation of the unfolded protein responses (UPR) are observed at all stages of atherosclerotic lesions, particularly in macrophages [1]–[3]. Lipid accumulation and disturbances in calcium homeostasis induce ER stress, the UPR and ER- associated degradation (ERAD) [4]–[6]. ER stress is an important event during the initiation, progression and clinical progression of atherosclerosis [3]. At an early stage of atherosclerosis, the increased number of apoptotic cells in macrophages suppresses early atherosclerotic lesion development [7]. ER stress-related proteins, such as glucose regulate protein 78 (GRP78) and C/EBP homologous protein (CHOP) are expressed in macrophage-derived foam cells [1]. At advanced stages, ER stress causes the apoptosis of macrophages, thus increasing the necrotic core size, and elicits inflammatory reactions [8].

The relationship between ER stress and inflammation has gradually been revealed. ER stress stimulates three distinct UPR-signaling pathways through sensors that include protein kinase-like ER kinase (PERK), inositol-requiring transmembrane kinase and endonuclease 1 (IRE1α) and activation of transcription factor 6 (ATF6). The PERK–CHOP pathway has been extensively investigated, and CHOP plays a central role in the inflammatory response and apoptosis of macrophages. CHOP deficiency prevents the development of atherosclerosis by reducing apoptosis and inflammation in the arteries of apoE^−/−^ mice [9]. It has been reported that the activation of NF-κB pathway occurs via the ATF-6 branch [10], [11].

Herp is an ER stress-associated protein that was originally found as a gene product that was upregulated in vascular endothelial cells treated with homocysteine [12]. It is ubiquitously expressed in various tissues and organs, and is highly expressed in the heart, liver, skeletal muscle, kidneys and pancreas [12]. Herp is dually regulated by shared (PERK/eIF-2alpha dependent) and the ER stress-specific (IRE1/XBP-1 and ATF6 dependent) mechanisms during UPR activation [13]. The protein plays a crucial role in the maintenance of calcium homeostasis during ER stress [14], [15]. It has been demonstrated that Herp has an ubiquitin-like domain at the cytoplasmic end, and it is considered to play a role in ERAD by recruiting ubiquitin [16], [17]. Herp has also been implicated in the pathogenesis of age-related disorders, including type 2 diabetes [18], neurodegeneration [19]–[21] and sarcopenia [22]. Herp-deficient neural cells accumulate more amyloid β-protein than wild type cells, and Herp-deficient muscle cells are more susceptible to ER stress-induced apoptosis [21], [22]. In spite of these diverse ER stress-associated functions, the role of Herp in the development of atherosclerosis is unknown. We therefore developed a Herp-deficient mouse model of atherosclerosis using apoE deficient mice, and studied whether Herp deficiency affects the development of atherosclerosis.

## Materials and Methods

### Materials

The anti-Herp antibodies have been described previously [23]. The anti-interleukin-1β (IL-1β), anti-glucose regulated protein 78 kDa (GRP78), normal rabbit IgG (Cell Signaling, Danvers, MA), anti-F4/80 (Abcam, Cambridge, UK) and anti-β-actin (Sigma, St. Louis, MO) antibodies were obtained from commercial sources. Tunicamycin, dimethyl sulfoxide (DMSO) (Sigma), fetal bovine serum (FBS) (HyClone, Logan, UT) and Dulbecco's modified Eagle medium (DMEM) (Nissui, Tokyo, Japan) were also commercially available.

### Animals

Herp^−/−^ mice were backcrossed for at least 10 generations with C57BL/6J mice (Japan SLC, Shizuoka, Japan) [23]. ApoE^−/−^ mice on the C57BL/6J background were purchased from The Jackson Laboratory (Bar Harbor, ME). To obtain Herp^−/−^; apoE^−/−^ mice, the crosses were set up as follows. Herp^−/−^ mice were crossed to apoE^−/−^ mice to obtain Herp^+/−^; apoE^+/−^ mice. The Herp^+/−^; apoE^+/−^ mice were intercrossed to produce Herp^−/−^; apoE^−/−^ mice. The mice were maintained on a normal diet after weaning. All procedures were performed in accordance with the guidelines for animal welfare of Tokyo Medical and Dental University, and the protocol was approved by the Animal Welfare Committee of the university.

### Quantitative analysis of arteriosclerotic lesions and blood chemistry

The atherosclerotic lesions in the aorta were analyzed as described previously [24]. Male apoE^−/−^ and Herp^−/−^; apoE^−/−^ mice were fed a normal diet, and the formation of atherosclerosis was studied at 16, 24, 36 and 72 weeks. After overnight fasting, the mice were anesthetized, a blood sample was drawn from the left ventricle and the entire aorta was resected. The aorta was opened longitudinally from the heart to the femoral arteries, pinned on a wax plate and fixed in 4% paraformaldehyde (Wako, Osaka, Japan). The aorta was photographed with an Olympus C-5050 ZOOM digital camera (Tokyo, Japan). The area of atherosclerotic lesions and the area of the entire aorta was determined using the NIH Image software program. The plasma levels of glucose, cholesterol, triglycerides and non-esterified fatty acid (NEFA) were determined with kits (Wako) as described previously [25].

### Histopathology

The aortas were fixed in the 4% paraformaldehyde or snap-frozen in liquid nitrogen. Serial thick sections were stained with hematoxylin-eosin and various antibodies. Briefly, paraffinized sections were deparaffinized, the endogenous peroxidase activity was blocked with 3% H_2_O_2_ in methanol for 30 min and the samples were stained with an anti-Herp (1∶100) antibody using an ABC elite kit (Vector Laboratories, Southfield, MI). Frozen sections were stained with anti-IL-1β (1∶100), anti-GRP78 (1∶100) or anti-F4/80 (1∶1000) antibodies using an ABC elite kit. Unless otherwise indicated, nonspecific immunostaining was not detected in sections stained with normal IgG as the primary antibody or with the secondary antibody alone.

### Cell culture

RAW264.7 cells, a mouse macrophage cell line, were obtained from Dainihon-Sumitomo pharmaceutical company (Osaka, Japan), and were cultured in DMEM supplemented with 10% FBS, 50 units/ml penicillin, 50 µg/ml streptomycin and 0.5 μg/ml amphotericin B. Peritoneal macrophages were obtained from eight-week-old apoE^−/−^ or Herp^−/−^; apoE^−/−^ mice. Briefly, 10 ml of sterile PBS were injected into the peritoneal cavity of the mice. The abdomen was gently massaged after its distension. The fluid was recovered and transferred to a sterile tube kept on ice. The cell suspension was centrifuged at 800× g for 5 min at 4°C. The pellet was washed once with chilled PBS and suspended in a volume of RPMI-1640 medium containing 10% FBS, 2 mM glutamine, 50 units/ml penicillin, 50 µg/ml streptomycin and 0.5 μg/ml amphotericin B. Cells were plated and incubated for 2 h at 37°C in a humidified CO_2_ (5%) incubator. The monolayers were washed with PBS three times to remove non-adherent cells, and monolayers were incubated overnight at 37°C in the above RPMI medium before being studied.

ER stress was induced by treatment with 1–10 μM tunicamycin (Sigma). The cell viability was measured by the 4-[3-(4-Iodophenyl)-2-(4-nitrophenyl)-2H-5-tetrazolio]-1, 3- benzene disulfonate (WST-1) assay (Roche Diagnostics, Basel, Switzerland).

### Immunoblotting

The immunoblot analysis of Herp in the aorta was conducted as reported previously [26]. The aorta was homogenized in ice-cold homogenization buffer (50 mM Tris-HCl, pH 8.0, 150 mM NaCl, 1% Triton-X100 and 1% protease inhibitor cocktail [Sigma]) with a physcotron NS-310E homogenizer (Microtec, Chiba, Japan). Proteins (10 μg) were run under reducing conditions on 5–20% gradient polyacrylamide gels and were transferred to PVDF membranes. The membrane was blocked in blocking buffer (5% fish gelatin and 0.5% Tween-20 in TBS) for 1 h, and then incubated with anti-Herp (1∶500) or anti-β-actin (1∶1000) antibodies in blocking buffer overnight at 4°C. After being washed with 0.5% Tween-20 in TBS, the membrane was incubated for 2 h with a HRP-conjugated secondary antibody and visualized using the ECL plus Western Blotting Detection System (GE Healthcare, Fairfield, CT).

### Transfection

Transfections of siRNA were performed with Lipofectamine 2000 (Invitrogen, Carlsbad, CA) as directed by the manufacturer. Briefly, cells were seeded at 2×10^5^ per well in six-well plates, then on the following day were transfected with 5 μl of Lipofectamine 2000 combined with 200 pmol of siRNA. The transfection mixtures were left on cells for 4 h, and then replaced with fresh media. At 48 h post-transfection, the cells were treated with tunicamycin (10 μM) or DMSO for 6 h. The siRNA against mouse Herp (sense sequence: 5′-UGG AUC ACC AGU GUC UCC AAG AUU U-3′, antisense sequence: 5′-AAA UCU UGG AGA CAC UGG UGA UCC A-3′) and negative control siRNA were obtained from Invitrogen.

### Real-time PCR analysis

Total RNA was prepared from each aorta using the RNeasy Mini kit (Qiagen, Duesseldorf, Germany). The first-strand cDNA was synthesized from 1 μg of total RNA using Omniscript Reverse Transcription (Qiagen). The real-time RT-PCR analyses were performed as described previously [27] using 10 ng of cDNA and TaqMan probes (Roche Diagnostics) for IL-1β, interleukin-6 (IL-6), monocyte chemoattractant protein-1 (MCP-1), stress-associated endoplasmic reticulum protein 1 (SERP1), Sec61b, sel-1 homolog 1 (SEL1L) and activating transcription factor 4 (ATF4) with a LightCycler instrument (Roche Diagnostics). The mRNA expression levels of Herp, GRP78, C/EBP homologous protein (CHOP), tumor necrosis factor-α (TNF-α, vascular cell adhesion molecule (VCAM) and glyceraldehyde-3- phosphate dehydrogenase (GAPDH), were evaluated as described previously with SYBR Green (Roche Diagnostics) [27]. The amount of target mRNA was normalized to that of GAPDH in the same sample. The RT-PCR for XBP1 was designed to amplify both the 140 bp (unspliced form) and 114 bp (spliced form) products [23]. The primers used for real-time PCR are listed in Table S1.

### Statistical analysis

The data were compared with a one-way ANOVA, followed by Scheffe's multiple comparison test or Student's *t* test. A value of P<0.05 was considered to be statistically significant. All values are expressed as the means ± SEM.

## Results

### Herp is expressed in the normal arteries

**Figure 1:**
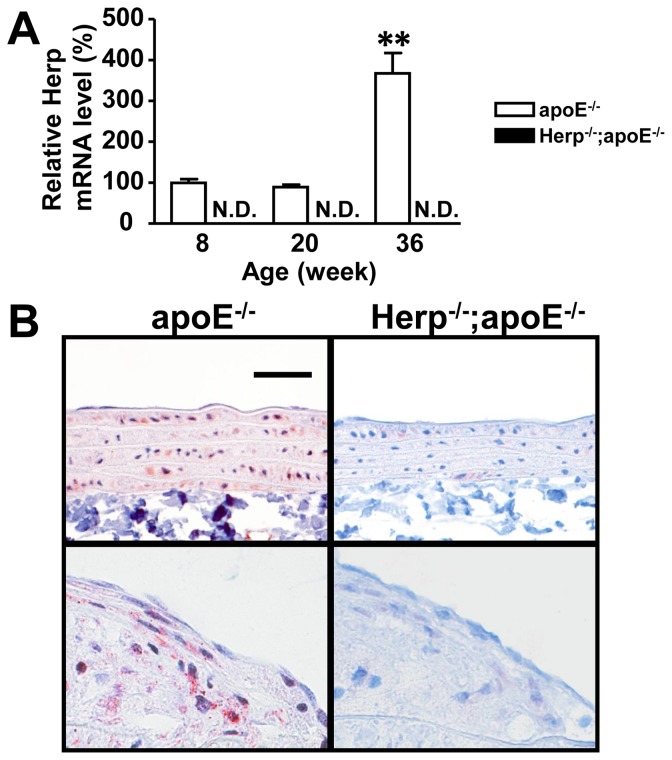
Herp expression in the artery. A: The amount for Herp mRNA was determined by a real-time PCR in the aortas of apoE^−/−^ mice (open columns) and Herp^−/−^; apoE^−/−^mice (closed columns). Each mRNA expression level was normalized to that of GAPDH. The mRNA levels are shown as relative ratios to the mRNA level of eight-week-old apoE^−/−^ mice. Each value represents the mean ± SEM of five mice. N.D. indicates non-detectable. ** p<0.01 vs. apoE^−/−^ mice at eight weeks of age. B: Immunostaining of Herp in the normal (upper panels) and atherosclerotic (lower panels) aortas of apoE^−/−^ and Herp^−/−^; apoE^−/−^ mice at 72 weeks of age. Blue; hematoxylin, Red; Herp (diaminobenzidine; DAB). The bar shows 50 μm.

To assess the role of Herp in the arteries, we first evaluated the expression levels of Herp in the arteries. Herp mRNA was detected in the aortas of apoE^−/−^ mice, but not in Herp^−/−^; apoE^−/−^ mice (Fig. 1A). The Herp mRNA expression was significantly increased in the older (36 weeks) mice (Fig. 1A). Immunostaining revealed that Herp was mainly expressed in smooth muscle cells. However, Herp was not detected in the endothelial cells in normal aorta (Fig. 1B). In atherosclerotic lesions, Herp was expressed in a portion of the macrophages (not in all macrophages), endothelial cells and smooth muscle cells (Fig. 1B, Fig. S1).

### The deficiency of Herp suppresses the development of atherosclerosis in apoE^−/−^ mice

**Figure 2:**
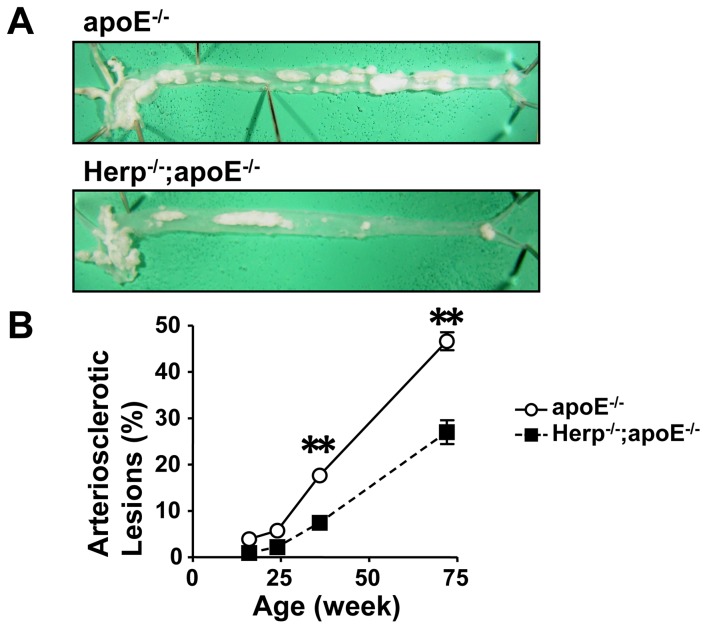
Herp deficiency suppressed the development of atherosclerosis in apoE^−/−^ mice. A: Photographs of the aortas of apoE^−/−^ (upper panel) and Herp^−/−^; apoE^−/−^ (lower panel) mice at 72 weeks of age. The aorta was opened longitudinally from the heart to the femoral arteries. The white plaques are atherosclerotic lesions. B: A quantitative analysis of the atherosclerotic lesions. The area was determined using the NIH Image software program. Each point represents the percentage of the lesion to the entire aorta. Open circles show apoE^−/−^, and closed squares show Herp^−/−^; apoE^−/−^ mice. Each value represents the mean ± SEM of four to six mice. ** p<0.01 vs. Herp^−/−^; apoE^−/−^ mice at the same age.

Next, we examined the effects of Herp on the development of atherosclerosis. The deficiency of Herp significantly suppressed the development of atherosclerosis in apoE^−/−^ mice (Figs. 2A, B). At 16 weeks, the apoE^−/−^ mice developed small fatty streaks at the aortic arch, whereas Herp^−/−^; apoE^−/−^ mice did not develop these lesions. At 24 weeks, the apoE^−/−^ mice developed sizable lesions at the arch, while Herp^−/−^; apoE^−/−^ mice had less developed lesions at the arch than apoE^−/−^ mice. At 36 weeks, the apoE^−/−^ mice had lesions in both the arch and abdominal aorta, while Herp^−/−^; apoE^−/−^ mice had atherosclerotic lesions only in the arch. At 72 weeks, the apoE^−/−^ mice showed atherosclerotic lesions that covered almost 50% of the entire aortic surface. In contrast, the Herp^−/−^; apoE^−/−^ mice had lesions that covered less than 30% of the surface (Figs. 2A, B).

### Herp deficiency affects the plasma glucose and lipid levels

**Figure 3:**
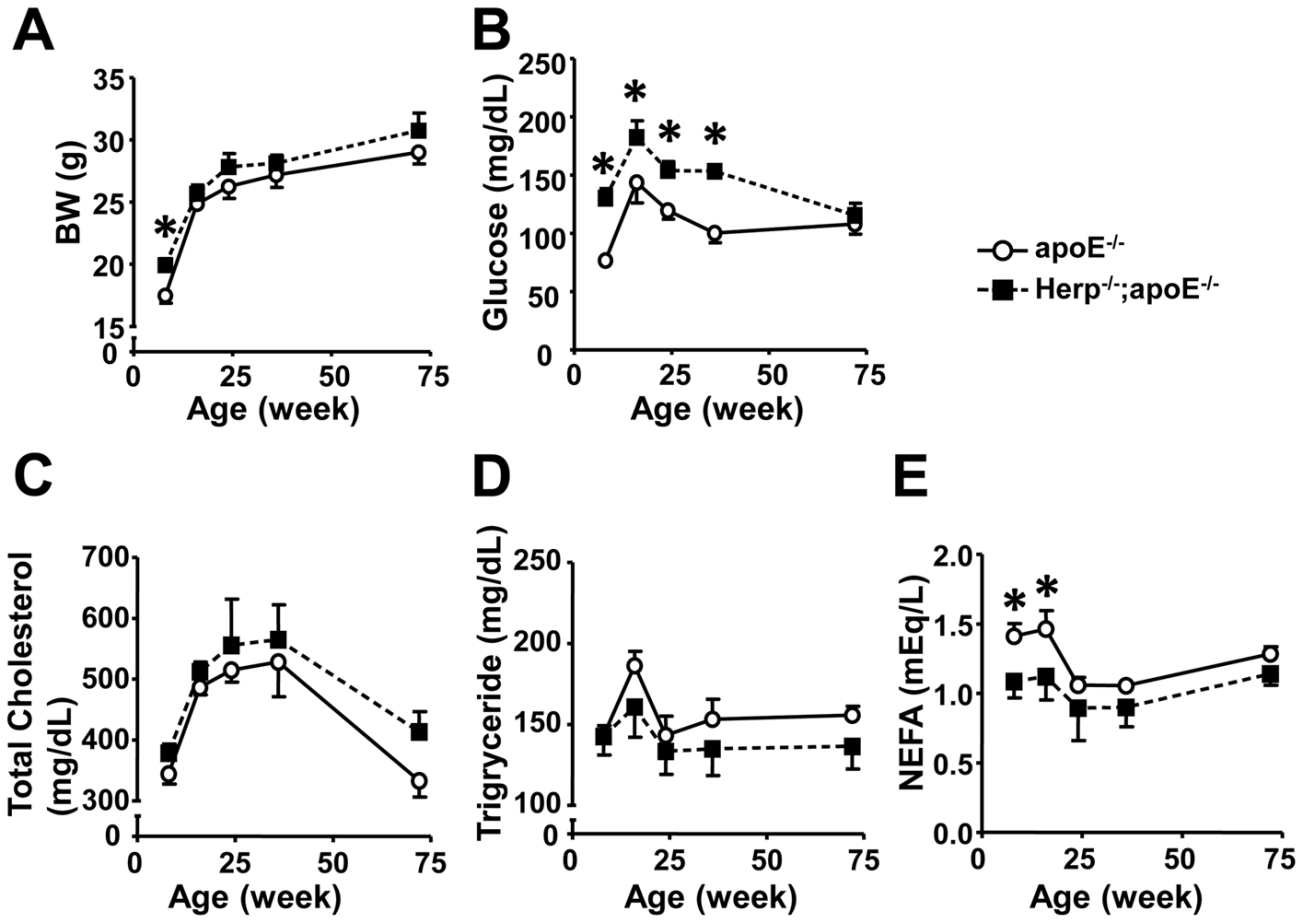
Effect of Herp deficiency on the plasma glucose and lipid levels. The body weight (A) and the plasma levels of glucose (B), cholesterol (C), triglycerides (D) and NEFA (E) following overnight fasting at eight, 16, 24, 36 and 72 weeks of age, were determined as described in the Methods. Open circles show apoE^−/−^, and closed squares show Herp^−/−^; apoE^−/−^ mice. Each value represents the mean ± SEM; n = 5 per group. * p<0.01 vs. Herp^−/−^; apoE^−/−^ mice at the same age.

The development of atherosclerosis is driven by high plasma levels of cholesterol [28], [29]. Since the response to ER stress results in lipogenic and cholesterogenic gene expression in hepatocytes [30], it is possible that Herp-deficiency affected the lesion formation by changing the plasma levels of lipids. We therefore determined the plasma levels of cholesterol, triglycerides and NEFA. The body weights were not significantly different between the apoE^−/−^ and Herp^−/−^;apoE^−/−^ mice (Fig. 3A). However, the levels of glucose were significantly higher in Herp^−/−^;apoE^−/−^ than in apoE^−/−^ mice at eight, 16, 24 and 36 weeks (Fig. 3B). There were no significant differences in the plasma levels of cholesterol and triglycerides between the Herp^−/−^; apoE^−/−^ and apoE^−/−^ mice (Figs. 3C, D). The plasma NEFA level was higher in apoE^−/−^ than Herp^−/−^; apoE^−/−^ mice at eight and 16 weeks, but not thereafter (Fig. 3E).

### Herp-deficiency attenuates the ER stress-induced unfolded protein response (UPR) and inflammation in the aorta

**Figure 4:**
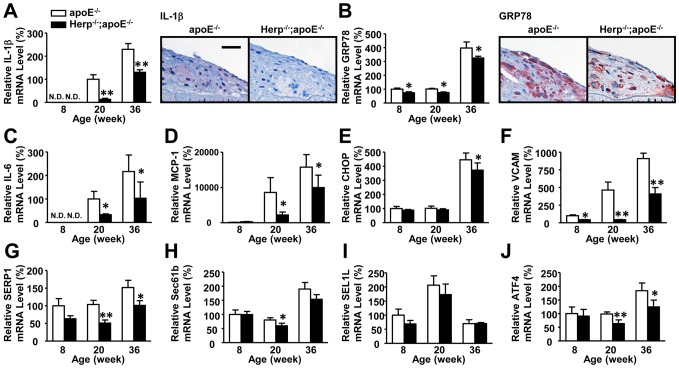
The expression levels of IL-1β, MCP-1 and VCAM-1 were reduced in the aortas of Herp^−/−^; apoE^−/−^ mice. Total RNA was prepared from the entire aortas of apoE^−/−^ and Herp^−/−^; apoE^−/−^ mice. The mRNA expression levels were determined by real-time PCR. Each mRNA level was normalized to that of GAPDH mRNA. The mRNA levels are shown as the relative ratio to the mRNA level of eight or 20-week-old apoE^−/−^ mice. A: IL-1β, B: GRP78, C: IL-6, D: MCP-1, E: CHOP, F: VCAM-1, G: SERP1, H: Sec61b, I: SEL1L and J: ATF4. Open columns show apoE^−/−^, and closed columns show Herp^−/−^; apoE^−/−^ mice. Each column represents the mean ± SEM of five mice. N.D. indicates non-detectable. * p<0.05, ** p<0.01 vs. apoE^−/−^ mice at the same age. A, B: Immunostaining of IL-1β and GRP78 in the atherosclerotic aortas of apoE^−/−^ and Herp^−/−^; apoE^−/−^ mice at 72 weeks of age. Blue; hematoxylin, Red; GRP78 or IL-1β (DAB). The bar shows 50 μm.

Activation of the UPR is observed at all stages of atherosclerotic lesion development in apoE^−/−^ mice [1]. We therefore assessed the UPR in the aortas of Herp-deficient mice. The amount of mRNA for inflammatory cytokines, such as IL-1β, IL-6 and MCP-1, was significantly lower in Herp^−/−^; apoE^−/−^ mice than in apoE^−/−^ mice (Figs. 4A, C, D). Among the various ER stress-related proteins, we found that the amount of mRNA for GRP78 was significantly lower in Herp^−/−^; apoE^−/−^ mice than in apoE^−/−^ mice (Fig. 4B), while that for CHOP showed a slight, but statistically significant, decrease in the Herp^−/−^; apoE^−/−^ mice (Fig. 4E). The amount of mRNA for VCAM-1 was also lower in Herp^−/−^; apoE^−/−^ mice (Fig. 4F). We also examined the expression of factors involved in other ER stress pathways, including IRE1, PERK and ATF6. The amount of mRNA for stress-associated endoplasmic reticulum protein 1 (SERP1) and Sec61b, an IRE1/XBP-1-dependent gene, was significantly decreased at 20 weeks in Herp^−/−^; apoE^−/−^ mice (Figs. 4G, H). The amount of mRNA for sel-1 homolog 1 (SEL1L) (Fig. 4I) and protein disulfide isomerase family A member 4 (PDIA4) (data not shown), ATF6-dependent genes, was not significantly different between apoE^−/−^ and Herp^−/−^; apoE^−/−^ mice (Fig. 4I). The amount of mRNA for activating transcription factor 4 (ATF4), a PERK-dependent gene, was significantly different at 20 and 32 weeks in Herp^−/−^; apoE^−/−^ mice (Fig. 4J) compared to the apoE^−/−^ mice.

An immunohistological analysis of the atherosclerotic lesions confirmed the results of the above-mentioned mRNA analyses. The IL-1β expression in macrophages was suppressed in atherosclerotic lesions of the Herp^−/−^; apoE^−/−^ mice (Fig. 4A). Consistent with previous reports [1], [31], GRP78 was extensively expressed in macrophage-derived foam cells of atherosclerotic lesions. The expression of the GRP78 protein was lower in Herp^−/−^; apoE^−/−^ mice (Fig. 4B).

### Herp-deficiency attenuates the tunicamycin-induced UPR and inflammatory responses in macrophages

An immunohistological study suggested that Herp-deficiency suppressed the ER stress responses, particularly in the macrophages in atherosclerotic lesions. We therefore studied the effects of Herp-deficiency in macrophages *in vitro*.

**Figure 5:**
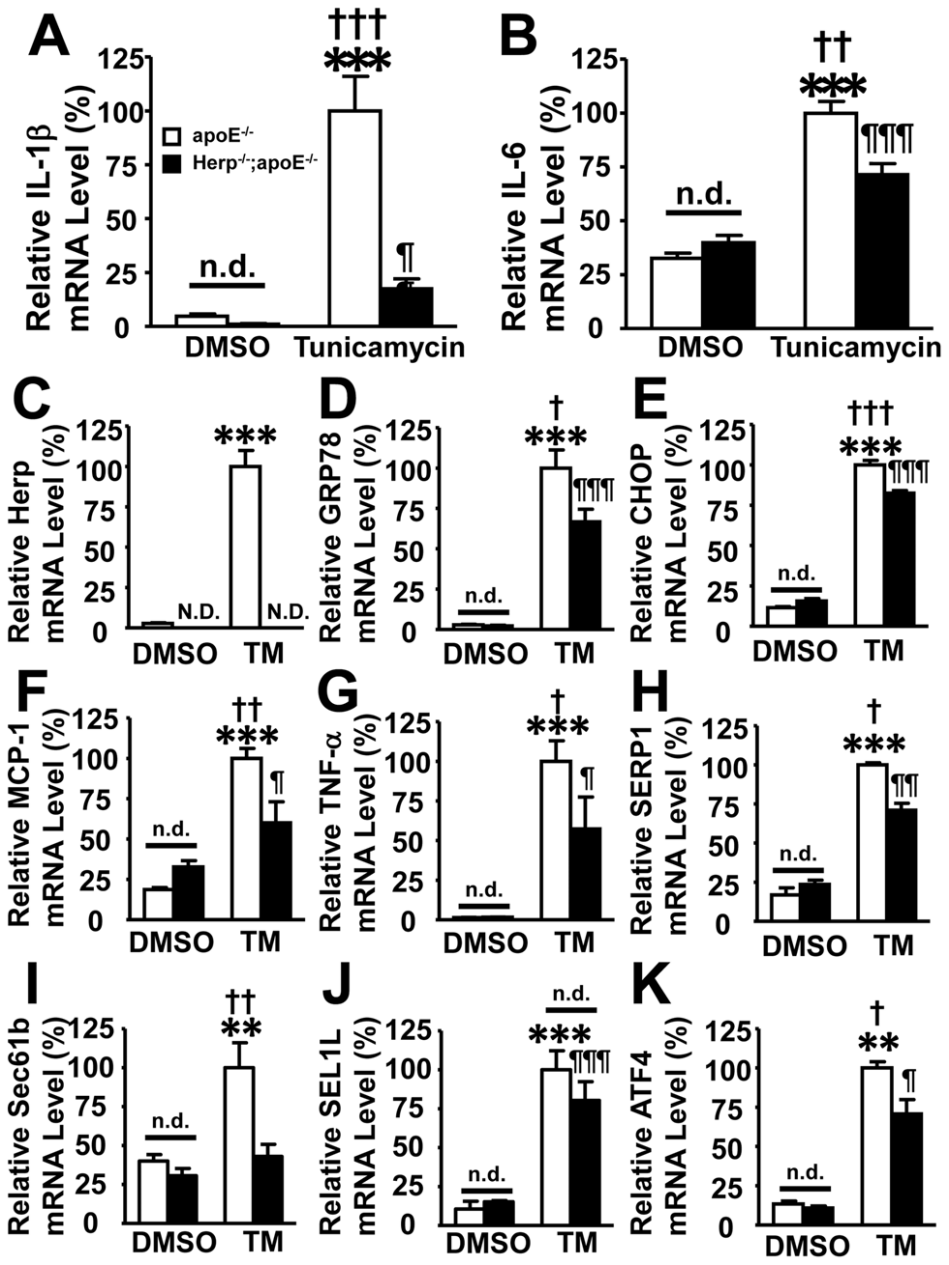
Herp-deficient peritoneal macrophages showed reduced expression of IL-1β and IL-6 in response to ER stress. Peritoneal macrophages were prepared from eight-week-old apoE^−/−^ and Herp^−/−^; apoE^−/−^ mice, and were stimulated with 10 μM tunicamycin for 6 hrs. The mRNA levels of ER stress-related proteins and cytokines were analyzed by a real-time PCR analysis. Open columns show unstimulated cells (DMSO), and closed columns show cells stimulated with tunicamycin (TM). Each amount of mRNA was normalized to GAPDH. The mRNA levels are shown as the relative ratio to the mRNA level of tunicamycin-treated macrophages from apoE^−/−^ mice. A: IL-1β, B: IL-6, C: Herp, D: GRP78, E: CHOP, F: MCP-1, G: TNF-α, H: SERP1, I: Sec61b, J: SEL1L and K: ATF4. Each column represents the mean ± SEM; n = 5 per group. ** p<0.01, *** p<0.001, vs unstimulated apoE^−/−^ macrophages; ¶p<0.05, ¶¶p<0.01, ¶¶¶p<0.001 vs unstimulated Herp^−/−^; apoE^−/−^ peritoneal macrophages. †p<0.05, ††p<0.01, †††p<0.001 vs stimulated Herp^−/−^; apoE^−/−^ peritoneal macrophages. N.D.; not detected. n.d.; no difference.

Consistent with previous reports [9], [32], inflammatory cytokines, such as IL-1β, IL-6, TNF-α and MCP-1, were induced in the macrophages of apoE^−/−^ mice. The induction of these cytokines was decreased in the macrophages from Herp^−/−^; apoE^−/−^ mice (Figs. 5A, B, F, G). The amount of mRNA for SERP1 and Sec61b was significantly decreased in tunicamycin-treated macrophages from Herp^−/−^; apoE^−/−^ mice compared to apoE^−/−^ mice (Figs. 5H, I). The amount of mRNA for SEL1L was not significantly different between apoE^−/−^ and Herp^−/−^; apoE^−/−^ mice (Fig. 5J). The amount of mRNA for ATF4 was significantly attenuated in tunicamycin-treated macrophages from Herp^−/−^; apoE^−/−^ mice compared with apoE^−/−^ mice (Fig. 5K).

Cultured peritoneal macrophages minimally expressed ER stress-related proteins, GRP78, CHOP and Herp, when they were unstimulated. However, once they were stimulated with tunicamycin, the peritoneal macrophages from apoE^−/−^ mice expressed GRP78, CHOP and Herp. In contrast, the peritoneal macrophages from Herp^−/−^; apoE^−/−^ mice expressed significantly less GRP78 and CHOP, and did not express Herp at all (Figs. 5C, D, E).

**Figure 6:**
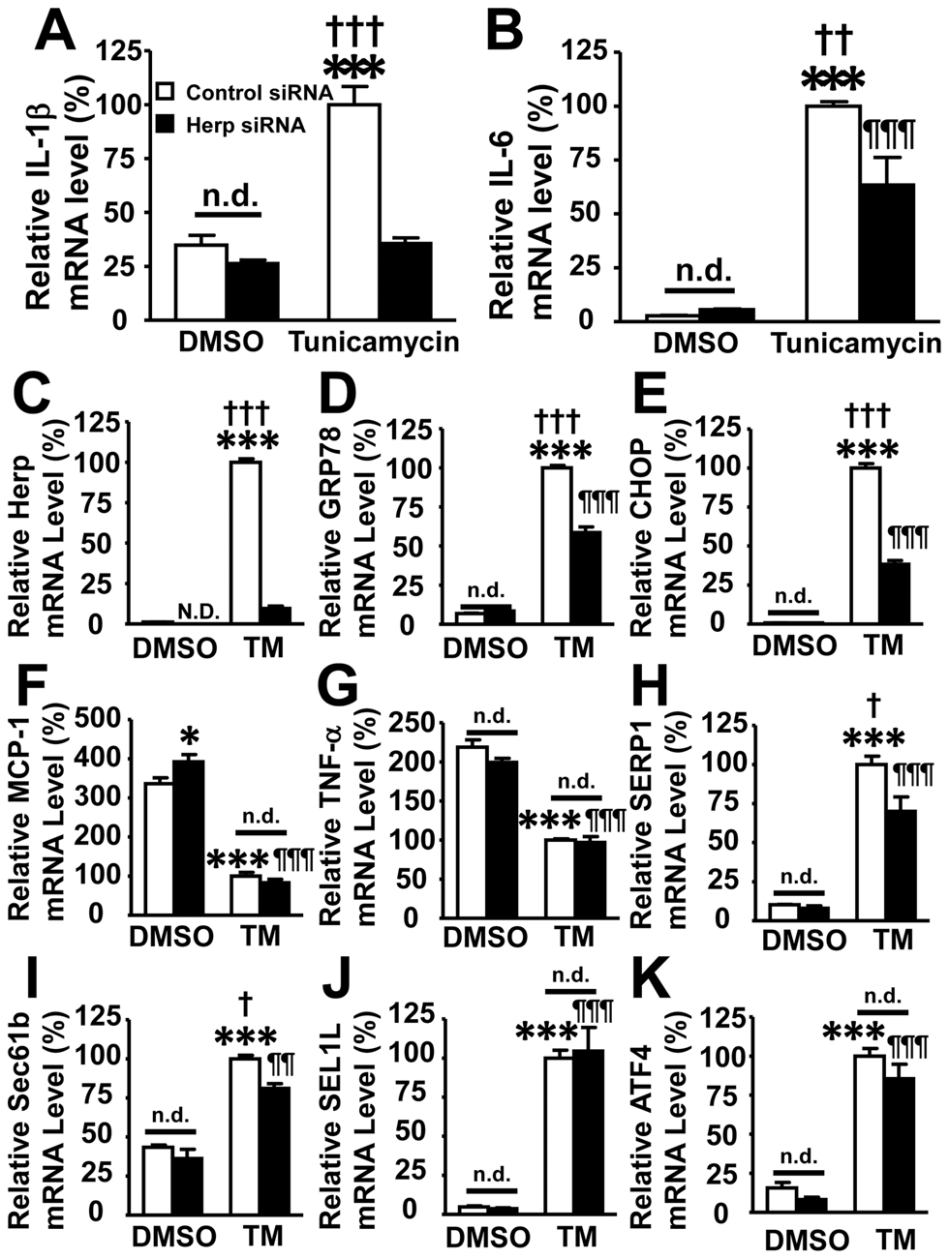
Herp-deficient RAW264.7 macrophages showed reduced expression of IL-1β and IL-6 in response to ER stress. RAW264.7 macrophages were treated with a siRNA against Herp or a control siRNA for 48 μM tunicamycin for 6 hrs. The mRNA expression levels of ER stress-related proteins and cytokines were analyzed by a real-time PCR analysis. Open columns show unstimulated cells (DMSO), closed columns show cells stimulated with tunicamycin (TM). The amounts of mRNA were normalized to that of GAPDH. The mRNA levels were shown as a relative ratio to the mRNA level of tunicamycin-treated macrophages, which were transfected with control siRNA. A: IL-1β, B: IL-6, C: Herp, D: GRP78, E: CHOP, F: MCP-1, G: TNF-α, H: SERP1, I: Sec61b, J: SEL1L and K: ATF4. Each column represents the mean ± SEM; n = 3 per group. * p<0.05, ** p<0.01, *** p<0.001, vs unstimulated control macrophages; ¶p<0.05, ¶¶p<0.01, ¶¶¶p<0.001 vs unstimulated siHerp macrophages. †p<0.05, ††p<0.01, †††p<0.001 vs stimulated siHerp macrophages. N.D.; not detected. n.d.; no difference.

The role of Herp in the ER stress response was further confirmed by treating RAW264.7 macrophages with a siRNA against Herp. Tunicamycin treatment induced the expression of ER stress response genes (such as GRP78 and CHOP) and Herp, and inflammation-related genes (such as IL-1β and IL-6) (Figs. 6A–E). The siRNA against Herp decreased the amount of Herp mRNA by 90% in RAW264.7 macrophages treated with tunicamycin, while the control siRNA did not affect the amount of Herp mRNA (Fig. 6C). Of note, the induction of GRP78, CHOP, IL-1β, IL-6, SERP1 and Sec61b was significantly suppressed by the treatment of the cells with the siRNA against Herp (Figs. 6A, B, D, E, H, I). However, there were no significant differences between control and siHerp-treated cells in terms of the ER stress-induced ATF4 mRNA expression (Fig. 6K). The mRNA levels of MCP-1 and TNF-α were decreased by tunicamycin treatment (Figs. 6F, G).

The mRNA expression of IRE1 dependent genes, such as SERP1 and Sec61b, was suppressed by Herp deficiency in macrophages from apoE^−/−^ and Herp^−/−^;apoE^−/−^ mice (Fig. 4) and RAW264.7 cells (Fig. 5). However, XBP-1 splicing was not prevented by Herp deficiency in macrophages and RAW264.7 cells (Fig. S2).

ER stress induces apoptosis in macrophages, which may suppress the development of atherosclerosis [33]. Tunicamycin caused a similar level of apoptosis in the peritoneal macrophages from both Herp^−/−^; apoE^−/−^ and apoE^−/−^ mice (Fig. S3).

## Discussion

A major novel finding of this study is that Herp deficiency suppressed the ER stress-induced inflammation and attenuated the development of atherosclerosis without affecting the plasma levels of cholesterol or triglyceride. Our findings indicate that Herp is also an essential component of ER stress-induced inflammation.

The precise mechanisms underlying the suppression of inflammatory cytokine production due to Herp deficiency are unclear. However, there are several possible mechanisms based on the functions of Herp that have been previously reported by others. First, recent reports have shown that these inflammatory cytokines are regulated by ER stress, primarily via the CHOP pathway [9], [34], [35]. CHOP deficiency also decreases the expression of inflammatory cytokines in arteries and suppresses arteriosclerosis in apoE-deficient mice [9]. Similar to CHOP deficiency, Herp deficiency suppresses ER stress-induced inflammation and attenuates the development of atherosclerosis. Our study showed that Herp deficiency decreases the expression of CHOP in the aortas. Herp may therefore regulate ER stress-induced inflammation by decreasing the CHOP expression. However, the effects of Herp deficiency cannot be attributed solely to a decrease in the CHOP expression [8] because CHOP deficiency, not Herp deficiency, decreases apoptosis among macrophages (Fig. S3). Herp deficiency might be more efficient for reducing the inflammatory response than for inducing the apoptosis of macrophages. Second, the suppression of IL-1β and TNF-α by Herp deficiency may involve the PERK – NF-κB pathway. The mRNA expression levels of molecules downstream of the PERK pathway, such as ATF4, were significantly attenuated by a Herp deficiency in the aorta and the tunicamycin-treated macrophages. The attenuated PERK-mediated reduction in the level of IκB results in a decreased NF-κB activation [36]. In contrast to ATF4, ATF6, another molecule that regulates NF-κB activation upon ER stress [10], [11], is not involved in the decrease in the mRNA expression of IL-1β and TNF-α in Herp-deficient mice. In this study, we showed that Herp deficiency does not affect the mRNA expression of SEL1L (Figs. 4I, 5J, 6J) or PID1A (data not shown), which are ATF6-dependent genes. Finally, the decrease in NEFA due to Herp deficiency may contribute to the suppression of inflammation in apoE-deficient mice. A deficiency of Herp caused a transient mild decrease in the plasma NEFA concentration at eight and 16 weeks in these mice (Fig. 3C). Since NEFA induces ER stress and inflammation, the decrease in NEFA may explain the attenuation of atherosclerosis in the Herp^−/−^;apoE^−/−^ mice. However, the lower level of NEFA may not be the major reason for the reduction in ER stress-induced inflammatory reactions because the change in the NEFA concentrations was mild, and a reduction in the inflammation-related gene expression due to Herp deficiency was observed in both the Herp-deleted peritoneal macrophages and RAW264.7 cells *in vitro*. Why the concentrations of NEFA were lower in the Herp^−/−^;apoE^−/−^ mice is not clear. A possible explanation is a reduction in the ER stress response in the adipose tissue. ER stress in adipocytes induces lipolysis by activating the cAMP/PKA and ERK1/2 pathways [37]. If the reduction of the ER stress response occurred in the adipose tissue of Herp^−/−^;apoE^−/−^ mice, the plasma levels of NEFA may be lower in Herp^−/−^;apoE^−/−^ mice than in apoE^−/−^ mice.

The induction of MCP-1 and TNF-α mRNA was attenuated by tunicamycin treatment in the macrophages collected from the Herp^−/−^; apoE^−/−^ mice (Figures 5F, G). However, the tunicamycin-treated RAW264.7 cells exhibited a decreased mRNA expression of MCP-1 and TNF-α, regardless of the presence or absence of Herp (Figure 6). These reductions were not observed in the RAW 264.7 cells possibly because the RAW264.7 cells were established by the inoculum of the moloney murine leukemia virus (MuLV) [38]. RAW264.7 cells also express polytropic MuLV [39], which may not possess functional p53 [40]. We therefore considered that these differences between the primary cultured macrophages and RAW264.7 cells were caused by the differences in the degree of activated p53. However, further studies are required to investigate these mechanisms.

We also showed that Herp has a unique expression pattern, wherein it is expressed not only in a small portion of macrophages in atherosclerotic lesions, but also smooth muscle cells (SMCs) in normal aorta (Fig. 1B). Of note, the expression levels of Herp are not upregulated at 20 weeks in apoE^−/−^ mice (Fig. 1A). The expression of Herp was not increased in the media that underlie the atherosclerotic plaques. In contrast, GRP78 was vigorously expressed in most macrophages in the lesions and increased in the lesions underlying the atherosclerotic plaques (Fig. 4G) [1], [31]. There are two major roles of Herp that have been speculated based on previous reports in other tissues. These include the secretion of proteins [41] and calcium homeostasis [2]. Herp plays a role in the ER stress response, likely due to the production of extracellular matrix proteins. And Herp may be expressed in the SMCs of normal aortas because it stabilizes the Ca^2+^ storage in the ER.

In summary, we herein reported a novel function of Herp in ER stress-induced inflammation, and described its potential role in the development of atherosclerosis.

## Supporting Information

Figure S1 **Herp was expressed in a subset of macrophages and smooth muscle cells in atherosclerotic lesions.** Immunostaining of the aorta from apoE^−/−^ mice. Blue; hematoxylin, Red; Herp (diaminobenzidine; DAB). The bar shows 50 μm.
(TIF)

Figure S2 **Herp deficiency did not increase the XBP-1 splicing in macrophages.** Peritoneal macrophages were prepared from apoE^−/−^ and Herp^−/−^; apoE^−/−^ mice, and stimulated with 1 to 10 μM tunicamycin for 6 hrs. The transfection of siHerp into RAW264.7 cells was described in the Methods section. RT-PCR for XBP1 was designed to amplify both the 140 bp (unspliced form) and 114 bp (spliced form) products.
(TIF)

Figure S3 **Herp deficiency did not increase the ER stress-induced apoptosis in macrophages.** Peritoneal macrophages were prepared from apoE^−/−^ and Herp^−/−^; apoE^−/−^ mice, and were stimulated with 1 to 10 μM tunicamycin for 6 hrs. The cell viability was determined as described in the Methods.
(TIF)

Table S1 **Real-time PCR and PCR primers.** The real-time PCR primers were designed to anneal to the indicated sequences using the free Primer3 software program (http://frodo.wi.mit.edu/cgi-bin/primer3/primer3_www.cgi).
(DOC)
