# Supplementary figures and images for: A Deficiency of Herp, an Endoplasmic Reticulum Stress Protein, Suppresses Atherosclerosis in ApoE Knockout Mice by Attenuating Inflammatory Responses
Source: PLoS One. 2013 Oct 28;8(10):e75249. doi: 10.1371/journal.pone.0075249 (PMC3810372; doi:10.1371/journal.pone.0075249)

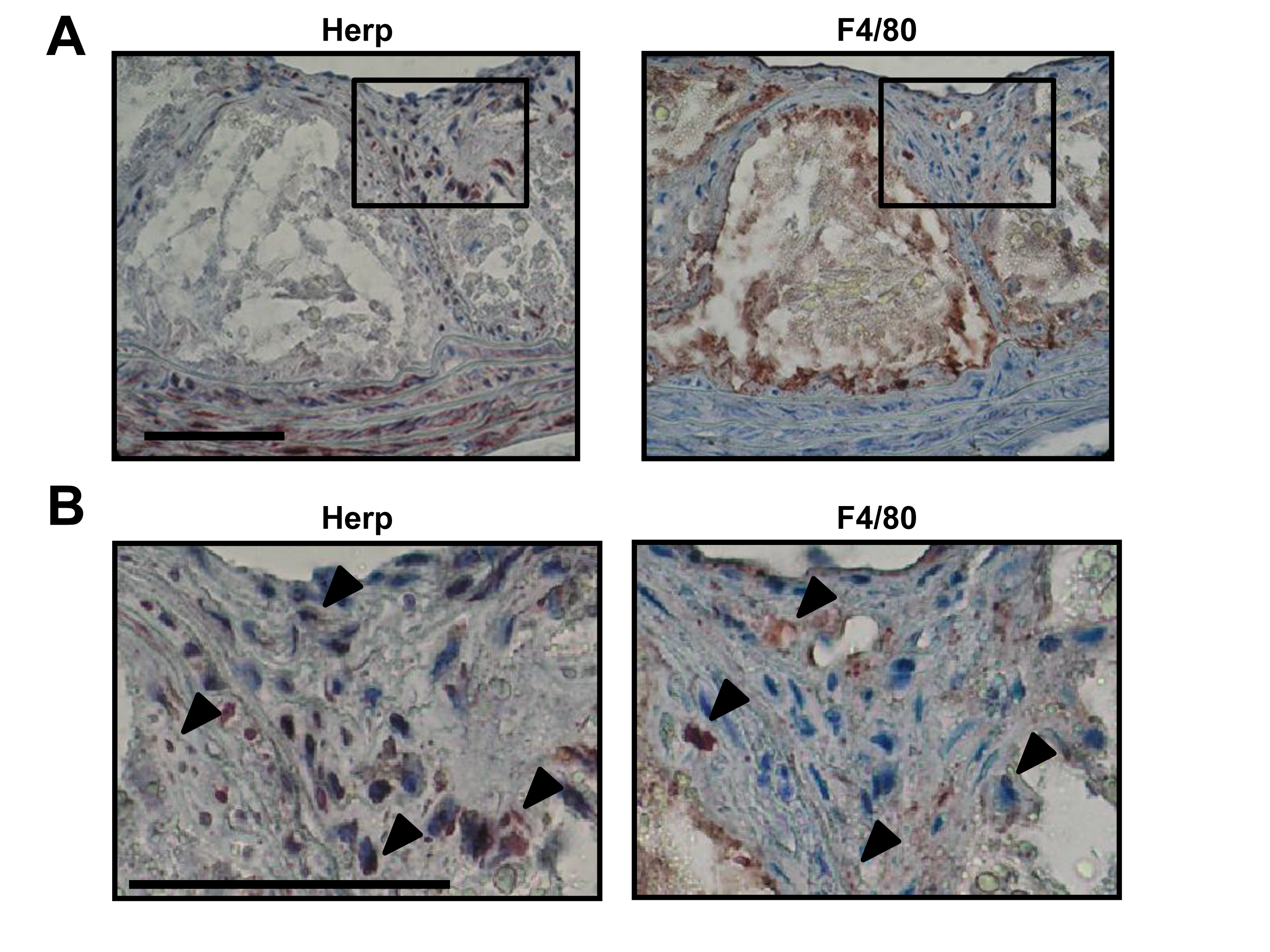

Supplement: Figure S1 — Herp was expressed in a subset of macrophages and smooth muscle cells in atherosclerotic lesions. Immunostaining of the aorta from apoE−/− mice. Blue; hematoxylin, Red; Herp (diaminobenzidine; DAB). The bar shows 50 μm. (TIF) [file pone.0075249.s001.tif]

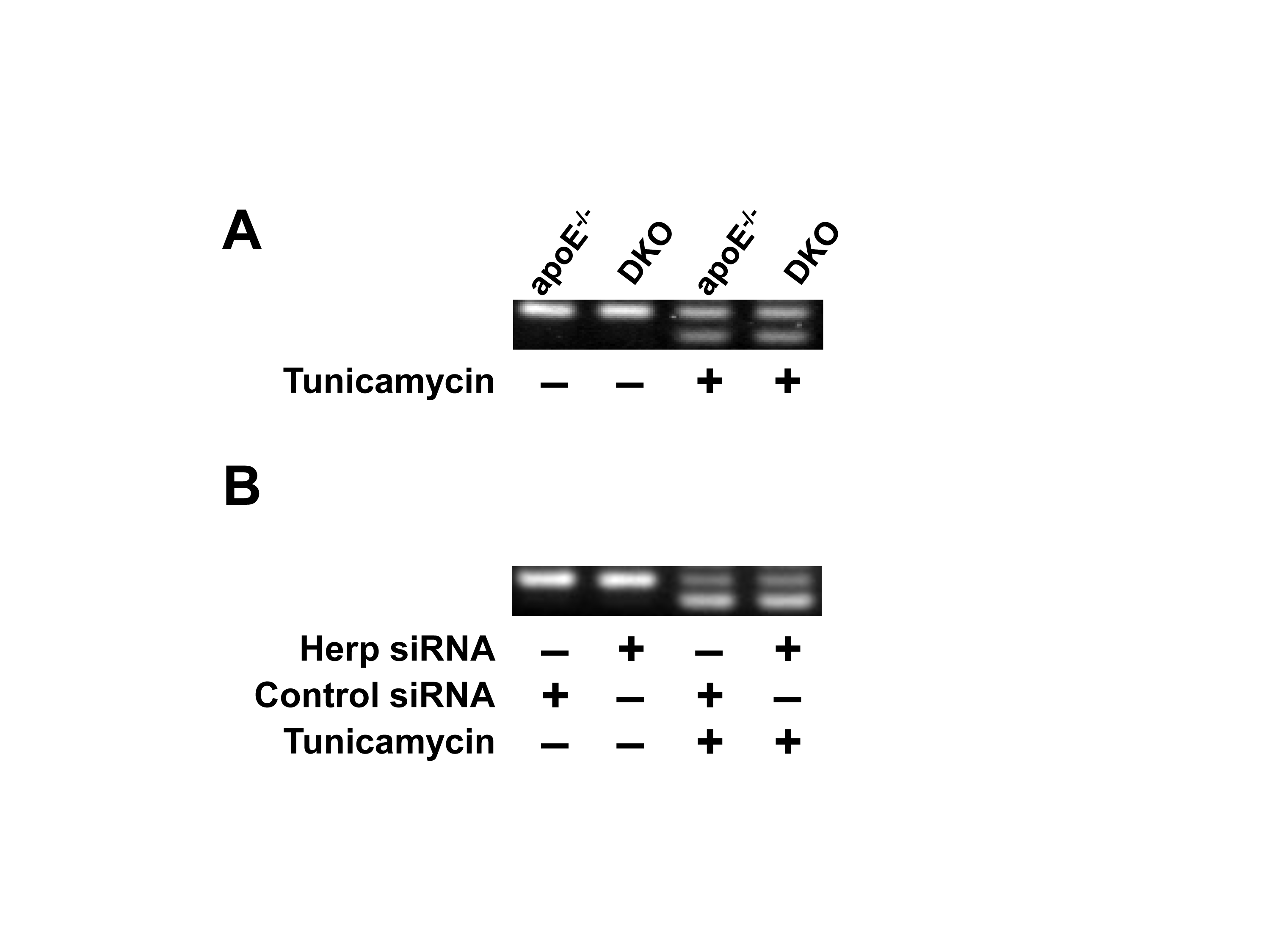

Supplement: Figure S2 — Herp deficiency did not increase the XBP-1 splicing in macrophages. Peritoneal macrophages were prepared from apoE−/− and Herp−/−; apoE−/− mice, and stimulated with 1 to 10 μM tunicamycin for 6 hrs. The transfection of siHerp into RAW264.7 cells was described in the Methods section. RT-PCR for XBP1 was designed to amplify both the 140 bp (unspliced form) and 114 bp (spliced form) products. (TIF) [file pone.0075249.s002.tif]

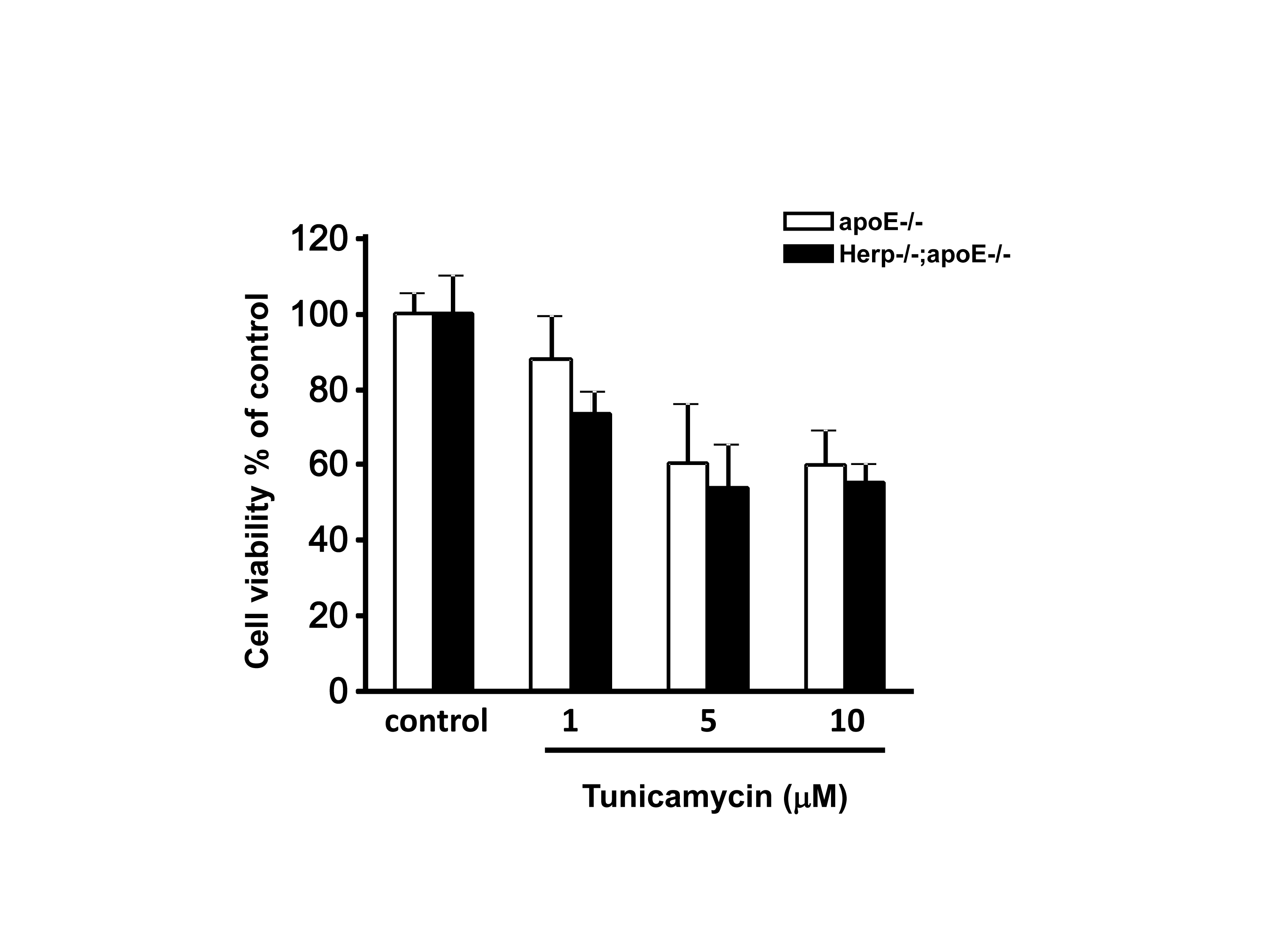

Supplement: Figure S3 — Herp deficiency did not increase the ER stress-induced apoptosis in macrophages. Peritoneal macrophages were prepared from apoE−/− and Herp−/−; apoE−/− mice, and were stimulated with 1 to 10 μM tunicamycin for 6 hrs. The cell viability was determined as described in the Methods. (TIF) [file pone.0075249.s003.tif]
