# Supplementary material for: A Deficiency of Herp, an Endoplasmic Reticulum Stress Protein, Suppresses Atherosclerosis in ApoE Knockout Mice by Attenuating Inflammatory Responses
Source: PLoS One. 2013 Oct 28;8(10):e75249. doi: 10.1371/journal.pone.0075249 (PMC3810372; doi:10.1371/journal.pone.0075249)
Supplement: Table S1 — Real-time PCR and PCR primers. The real-time PCR primers were designed to anneal to the indicated sequences using the free Primer3 software program (http://frodo.wi.mit.edu/cgi-bin/primer3/primer3_www.cgi). (DOC) [file pone.0075249.s004.doc]

**Table S1.**

| GenBank ID | Gene | Forward primer (5'-3') | Reverse primer (5'-3') | Product size (bp) |
| --- | --- | --- | --- | --- |
| X67083 | CHOP | cctagcttggctgacagagg | ctgctccttctccttcatgc | 196 |
| BC091768 | GAPDH | CTCATGACCACAGTCCATGC | CACATTGGGGGTAGGAACAC | 201 |
| D78645 | GRP78 | tggagttccccagattgaag | gcgctctttgagctttttgt | 199 |
| AB034991 | Herp | ATCAGAACTTGCGGATGAATG | GTTATTGTTGGGGTCCTCCTGGTT | 284 |
| AB301555 | IL-1β | CTGTGTTTCCTCCTTGCCTCTG | GCTGCCTAATGTCCCCTTGAAT | 104 |
| M20572 | IL-6 | TGCTGACCTCTGGACGCTTACT | GAGACCCATGCCTAACAACTCC | 123 |
| BC027520 | MCP-1 | CATCCACGTGTTGGCTCA | GATCATCTTGCTGGTGAATGAGT | 76 |
| D84199 | TNF-α | ACAGAAAGCATGATCCGCG | AAGAGGCTGAGACATAGGCACC | 101 |
| X67783 | VCAM-1 | attttctggggcaggaagtt | acgtcagaacaaccgaatcc | 238 |
| AF027963 | XBP-1 | GATCCTGACGAGGTTCCAGAG | AAGATGTTCTGGGGAGGTGAC | 140, 114 |
